# How Tea Consumption Interacts with Genetics to Affect Cardiovascular Biomarkers in Older Adults? Findings from the CLHLS

**DOI:** 10.21203/rs.3.rs-7554829/v1

**Published:** 2025-10-24

**Authors:** Jiali Wang, Kaisy Xinhong Ye, Changjiang Li, Min Zou, Luwen Cao, Xiu Wang, Lirong Yu, Lina Sun, Andrea B. Maier, Yanyu Wang, Juntang Guo, Yi Zeng, Huashuai Chen, Qiushi Feng

**Affiliations:** Shandong Second Medical University; National University of Singapore; Shandong Second Medical University; Shandong Second Medical University; University of Singapore; Beijing Chuiyangliu Hospital; Shandong Second Medical University; Shandong Second Medical University; National University of Singapore; Shandong Second Medical University; Shandong Second Medical University; National School of Development, Peking University; Duke University; National University of Singapore

**Keywords:** CVD, tea, tea consumption, polygenetic risk, gene-tea interaction, older adults, China

## Abstract

**Background/Objectives:**

Cardiovascular disease (CVD) has become the leading cause of mortality and disability worldwide. Tea is one of the most common consumed beverages worldwide. Studies have demonstrated that tea consumption has an impact on cardiovascular diseases. This study aims to examine the association between tea consumption and CVD biomarkers and investigate the potential effect of genetic variants on the relationship between tea consumption and CVD biomarkers in older adults;

**Methods:**

This prospective cohort study using four waves (2008, 2011, 2014, and 2018) of data from the Chinese Longitudinal Healthy Longevity Survey (CLHLS) to analyze the interactive effect of tea consumption and genetics on CVD biomarkers. The study sample consisted of older adults aged 65–105 from 8 longevity counties across China. The frequency and type of tea consumption and various covariates were investigated using questionnaires, seven blood biomarkers including Plasma glucose (PG),Total cholesterol(TC),Triglyceride(TG),High-sensitive C-reactive protein, (hs-CRP),High-density lipoprotein cholesterol(HDLC),Low-density lipoprotein cholesterol(LDLC) and Hemoglobin were selected from blood biochemical test, genetics were measured using the polygenic risk score(PRS) calculated by PLINK1. Generalized estimation equations (GEE) with a identity link function were adopted to estimate the effect of tea consumption and PRS on CVD biomarkers from both cross-sectional and longitudinal perspective;

**Results:**

The results showed that the consumption of tea was associated with higher levels of TC, TG, and LDLC levels, lower levels of HDLC, and age-specific effects on hs-CRP levels, and drinking tea increased TG and LDLC levels of higher PRS older adults, while lowering their HDLC and hs-CRP levels.

**Conclusions:**

The current study found that tea consumption had both beneficial and unfavorable effects on cardiovascular biomarkers in the older population. Specifically, it is protective against inflammatory related biomarkers and harmful for lipid metabolism related biomarkers, and these effects were all modified by genetic variants affecting lipid and inflammation metabolism.

## 1. Introduction

The world’s population is aging rapidly due to the low birth rate and prolonged life expectancy. Aging is a predominant risk factor for most chronic diseases such as cardiovascular diseases, cancer, and neurodegenerative diseases[1, 2]. Cardiovascular disease (CVD) has become the leading cause of mortality and disability worldwide. In China, the number of people with CVD reached about 330 million in 2022 and has been continuously increasing[3]. The primary prevention of CVD has become a critical public health concern.

Modifying lifestyle-related factors is an essential approach to the management of CVD[4]. Amongst numerous lifestyle factors related to CVD, tea consumption has been long suggested protective against the development of CVD [4–8]. A body of meta-analysis and high-controlled human research recognized that tea consumption is associated with risk factors of CVD, such as reductions in blood pressure[9], improvements in endothelial function[10], anti-arrhythmic[11], and reduced risk of ischemic heart disease[12]. Both human and animal studies have suggested that such a protective role of tea consumption are due to various bioactive compounds in tea leaves, such as tea catechins, theaflavins, and caffeine, which are recognized as natural antioxidants and anti-inflammatory agents, and play an essential role in antiatherogenic and antithrombotic properties through inhibiting lipoprotein oxidation, reducing vascular inflammation and regulating vascular reactivity [13–16].

However, in this field, studies on the relationship between tea consumption and classic biomarkers of CVD are still inconclusive, with mixed findings. Two studies from the UK Biobank and Dongfeng-Tongji cohort, for example, found that higher tea consumption was positively associated with lower total cholesterol(TC) and low-density lipoprotein cholesterol(LDLC) and higher high-density lipoprotein cholesterol(HDLC) [5,6], as are not in line with reports from a few randomized controlled trials (RCTs)[17–19]. This inconsistency, though possibly related to distinct research designs in age groups, sample sizes, trial durations, tea types and doses, and individual genetic makeup[6, 20, 21], clearly granted further investigations.

More importantly, this line of research should incorporate the mechanisms of gene-diet interactions, which has recently been highlighted as an important perspective with implications for the development of more targeted strategies in disease prevention and treatment[22]. It is evidenced that the biological effect of polyphenols on cardiovascular health is mediated by their capacity to modulate the expression of related genes[23–25]. Interestingly, some research also proposed otherwise, that the association between tea consumption and cardiovascular-related biomarkers was not modified by genetics[6]. Up to date, research on this topic is rare and mostly based on Western populations[26].

This study aims to investigate the association between tea consumption and CVD biomarkers among Chinese older adults. We used a large sample of older Chinese aged 65–105 from a national longitudinal survey. In this analysis, we further introduced the potential interaction between tea consumption and genetics to deepen understanding of the effect of tea consumption on CVD.

## 2. Materials and Methods

### 2.1 Participants

The study sample consisted of older adults from 8 longevity counties across China: Zhongxiang from Hunan Province, Mayang from Hunan Province, Chengmai from Hainan Province, Yongfu from Guangxi Province, Sanshui from Guangdong Province, Rudong from Jiangsu Province, Xiayi from Henan Province and Laizhou from Shangdong Province. The data were collected in the 2008, 2011, 2014, and 2018 waves of the Chinese Longitudinal Healthy Longevity Study (CLHLS).

The CLHLS is a large national longitudinal survey dated to the year 1998, which conducted home-based interviews on Chinese older adults aged over 65 across 23 provinces of China[27]. Since 2008, the CLHLS started to collect data from the selected counties of China as longevity areas designated by the Chinese National Research Center on Aging in terms of holding an exceptionally high density of centenarians. In 2008, 2011, 2014, and 2018 waves of the CLHLS, 1853, 2439, 2546, and 2567 participants were interviewed by staff of the China Center for Disease Control & Prevention (CDC), respectively. As a longitudinal investigation, the interviewed participants were tracked in the follow-up waves.

In this study, all 4 waves of CLHLS data from 2008 to 2018 are merged into an unbalanced panel data. Table 1 lists the characteristics of the study samples of CLHLS datasets. All participants were informed of the study’s purpose, confidentiality issues, and their rights as research participants.

### 2.2 DNA samples

The methodology of the CLHLS’s DNA sample was described in previous study[28]. This study pooled the DNA samples of the CLHLS from the 8 longevity areas in the 2008, 2011, and 2014 waves, obtaining a total of 3,694 DNA samples. After dropping those without complete information of DNA or cardiometabolic biomarkers, the final sample size was 3,200. To adopt the GWAS methods, we further distinguish two subsamples, one for discovery and the other for replication. Given the distribution of genetic characteristics of samples living in North China differ from in South China[29], we adopted a stratification framework with 1544 from South China as discovery samples and 1656 from North China as replication samples.

### 2.3 Tea consumption assessments and covariates

The CLHLS questionnaire asked about the frequency of drinking tea, which was classified as (1) Almost every day, (2) Occasionally (weekly/monthly/sometimes), and (3) Rarely/never. It further examined the type of tea consumed with two possible operationalization: Classification 1 with 5 types: (1) green tea, (2) oolong/white/yellow tea, (3) red/dark/compressed tea, (4) scented tea, (5) Not tea drinker; and Classification 2 in 3 types: (1) green tea, (2) not-green tea, (3) Not tea drinker. Note that information of types of tea consumption were reported only in the 2014 and 2018 waves of CLHLS. In order to assess both the current and historical status of tea consumption, the measures above were asked at two time points, at present and at around age 60.

Covariates in this study included age, gender (male or female), education (illiterate, elementary school, and middle school or higher), urban/rural residence, region (East provinces or Middle/West provinces), current marital status (yes or no), and living arrangements (live with family members, living alone or institutionalized), ever smoking (yes or no), alcohol consumption (yes or no), regular exercise (yes or no), and family income per capita, etc.

### 2.3 Serum cardiometabolic biomarkers

Non-fasting venous blood sampling was conducted in a standardized blood biochemical test procedure. Based on previous studies[5,6],we selected 7 biomarkers: Plasma glucose, mmol/L(PG),Total cholesterol, mmol/L(TC), Triglyceride, mmol/L (TG),High-sensitive c reactive protein, mg/l (hs-CRP),High-density lipoprotein cholesterol, mmol/L (HDLC),Low-density lipoprotein cholesterol, mmol/L(LDLC) and Hemoglobin, g/L.

For longitudinal analyses, we further obtained the cross-wave change of cardiometabolic biomarkers by comparing values of two adjacent waves. Note that the information on LDLC was only collected in the 2008 and 2011 waves of CLHLS. As data on the type of tea consumption was only available in the 2014 and 2018 waves, the association between types of tea consumption and LDLC cannot be analyzed.

### 2.4 Statistical analysis

We firstly summarize the sample characteristics and then used the Generalized estimation equation (GEE) to estimate the cross-sectional and longitudinal effect of tea consumption on CVD biomarkers with the stratified analyses by gender and age groups (65–79, 80–89 and 90–105). For the cross-sectional analyses, we modeled the cardiometabolic biomarkers and the tea consumption variables at the same wave; whereas for the longitudinal analyses, we instead focus on changes of cardiometabolic biomarkers during the 2008–2011, 2011–2014, and 2014–2018 intervals. In the GEE, we applied the identity link function and reported the coefficients and 95% confidence intervals (CIs) obtained from the model estimated robust standard errors. Exchangeable correlation structure was used to account for subject level repeated measures due to the survey design of the CLHLS. The analyses above was performed using Stata 17.1

To study the interaction effects with tea drinking behaviors on cardiometabolic biomarkers, we calculated Polygenic risk scores (PRS) by PLINK1 based on the discovery samples, using the --score option, which computes a linear function of the additively coded number of reference alleles weighted by the coefficients (betas). To pick up independent loci to construct PRS, we firstly removed those SNPs which do not have significant interaction effects with tea drinking behavior at present on cardiometabolic biomarkers in the discovery dataset. Among the total 287,898 imputed SNPs, we obtained 15494, 2032, 13340 and 16422 SNPs which have interaction effects with tea drinking on the 4 cardiometabolic biomarkers HDLC, LDLC, hs-CRP, TG, respectively, at P < 0.05. We standardized the PRS for each of the exclusive groups of the identified loci associated with cardiometabolic biomarkers at different significance levels through the z-scores transformation.

Lastly, we evaluated the associations between tea drinking behaviors (at present or around age 60), polygenic risk scores and the values of cardiometabolic biomarkers using OLS regression models in the Stata 17.1.

## 3. Results

### 3.1 Sample characteristics

Table 1 presents the characteristics of the study samples. The proportion of older people who drank tea daily or occasionally was around 20–40% in China. Specifically, the proportion of those who drank tea everyday ranges from 13.3% to 20.5% at present and 12.1% to 19.8% at around age 60. Regarding the tea type, the number of people who drink green tea was more than not-green tea at present, whereas the number of people who drink not-green tea was more than green tea at around age 60.

### 3.2 Cross-sectional association of tea consumption frequencies with CVD biomarkers

We used GEE to estimate the cross-sectional associations of frequencies of tea consumption with cardiometabolic biomarkers for two time points, for tea drinking at around age 60 (Table 2–1) and at present (Table 2–2), respectively. As shown, everyday tea drinking was significantly associated with higher TG levels, both for at around age 60(β = 0.07, 95%CI = 0.02–0.13, p < 0.01) and at present (β = 0.07, 95%CI = 0.02–0.11, p < 0.01). Everyday tea drinking at present was significantly associated with higher TC (β = 0.17, 95% Cl = 0.00–0.34, p < 0.05), but not for at around age 60. Similarly, everyday tea drink-ing at present was significantly associated with higher HDLC, but not for at around age 60. Everyday tea drinking at present was significantly associated with higher LDLC levels. Occasionally tea drinking at present significantly associated with higher LDLC levels, and occasionally tea drinking at around age 60 was significantly associated with higher LDLC levels. Occasionally tea drinking at around age 60 was also significantly associated with higher Hemoglobin levels, both for at present and at around age 60. Regarding PG and hs-CRP, no significant associations were found for the whole sample.

Age-stratified analyses revealed that everyday tea drinking at present was asso-ciated with higher level of HDLC (β = 0.05,95%CI = 0.00–0.09, p < 0.05) in age 80–89, not in age 65–79 and age 90–105. It was also significantly associated with higher LDLC levels in age 65–79 and 80–89 but not in age 90–105. This pattern is also observed in TG levels. Occasionally tea drinking at present was positively associated with PG levels in age 65–79 group, but not in age 80–89 and age 90–105. In age 65–79, everyday tea drinking at around age 60 was negatively associated with Hemoglobin levels but not in age 80–89 and 90–105. Gender-stratified analyses further showed that the associations between everyday tea drinking, both at present and at around age 60, and higher TG levels were only significant in males. This applies to the associations between occasionally tea drinking and LDLC levels as well. Occasionally tea drinking at present was signifi-cantly associated with higher Hemoglobin in females, however, every day tea drinking at 60 was negatively associated with Hemoglobin levels in females; every day tea drinking at present was associated with hs-CRP in females (β = 0.03, 95%CI = 0.00–0.07, p < 0.05),no significant results were found in males.

### 3.3 Cross-sectional association of types of tea consumption with CVD biomarkers

We further examined the cross-sectional associations of types of tea consumption with cardiometabolic biomarkers, for tea drinking at around age 60 (Table 3–1) and at present (Table 3–2), respectively. As showed in Table 3–1, green tea drinking at around 60 was associated with higher TC levels in age 90–105 (β = 0.22, 95%CI = 0.03–0.42, p < 0.05) as well as lower Hemoglobin levels in males (β =−5.28, 95%CI = −8.61–1.94, p < 0.05). White tea drinking at around age 60 was negatively associated with PG levels in age 90–105 groups (β=−0.44, 95%CI =−0.80–0.09, p < 0.05). Scented tea drinking at around age 60 was significantly associated with higher PG levels in age 90–105, lower hs-CRP levels in age 80–89, lower HDLC levels in age 90–105 and higher Hemoglobin levels in age 80–89. Table 3–2 further showed that, green tea drinking at present was significantly associated with higher PG in age 65–79, higher TC in age 90–105 and lower Hemoglobin in males. White tea drinking at present was significantly associated with higher HDLC in age 90–105. Black tea drinking at present was significantly associated with lower hs-CRP levels in females and age 90–105, and higher Hemoglobin levels in both male and female and in age 80–89.

### 3.4 Longitudinal association of frequencies of tea consumption with CVD bi-omarkers

Next, we investigated the longitudinal associations between frequencies of tea consumption and cardiometabolic biomarkers changes during the 2008–2011, 2011–2014, and 2014–2018 intervals, for tea drinking at around age 60 (Table 4–1) and at present (Table 4–2). As revealed, everyday tea drinking at present was negatively associated with Hemoglobin changes in females (β=−2.71, 95%Cl =−4.83–0.60, p < 0.05). Tea drinking at around age 60 was negatively associated with HDLC changes (β=−0.03, 95%CI=−0.06–0.00, p < 0.05), which was only observed in males, age 65–79 and age 80–89. Occasionally tea drinking at around age 60 was positively associated with hs-CRP changes in age 65–79, negatively associated in age 80–89, and not associated in age 90–105.

### 3.5 Interaction between genetics and tea consumption on CVD biomarkers

**Figure 1:**
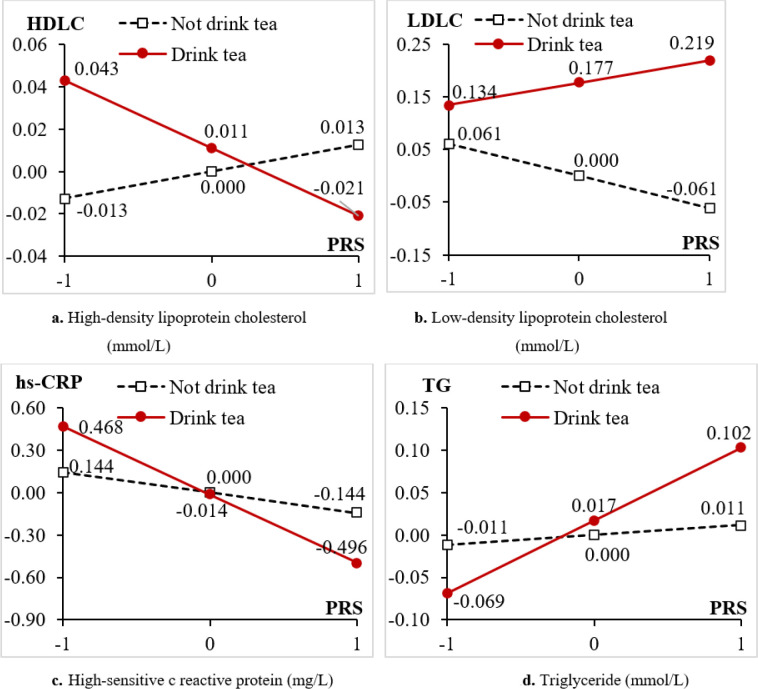
Comparison of Coefficients of PRS on cardiometabolic biomarkers by Tea drinkers and Non-tea-drinkers at present

Using OLS models, we examined the interaction effects between PRS and tea consumption on cardiometabolic biomarkers. We found significant interaction effects of tea drinking and PRS on four CVD biomarkers, namely HDLC, LDLC, hs-CPR and TG. Figure 1 illustrated the patterns of these interaction effects between PRS and tea consumption at present and at around age 60 in the whole sample. For gender and age stratified results, please refer to appendix A.

## 4. Discussion

This study used the four waves of CLHLS data to explore both the cross-sectional and longitudinal relationship between tea consumption and 7 CVD related biomarkers in older adults in China aged 65–105, and further. Additionally, it investigated the in-teraction effects of genetics and tea consumption on CVD related biomarkers. Major results in this study showed that the consumption of tea was associated with higher levels of TC, TG, and LDLC, lower levels of HDLC, and age-specific effects on hs-CRP levels; and drinking tea increased TG and LDLC levels of higher PRS older adults, while lowering their HDLC and hs-CRP levels. The study suggested that the associa-tion between frequency and type of tea consumption with CVD biomarkers is valid, varying by gender, age group and genetic variants, but could be mixed with beneficial effects on inflammatory-related biomarkers and proliferation-related biomarkers, and unfavorable effects on lipid-related biomarkers.

In the cross-sectional analyses, we found that the frequency of drinking tea was associated with higher levels of TC, TG, and LD-C, whereas in the longitudinal anal-yses, it was associated with lower levels of HDLC. This is not entirely consistent with some previous studies, which reported favorable affects of tea drinking on lipid metabolism[30–33]. We thus consider this study opening a door for discussions of future studies, but propose the effect of survival bias could be relevant for this in-consistency. The relationship between low blood lipid levels and the reduced risk of cardiovascular disease is well established. Yet some prospective cohort studies in older adults on the relationship between all-cause mortality and lipid profiles showed that higher TC, LDL-C and TG levels were significantly associated with lower risk of all-cause mortality in the older population[34–36]. Using the same data, we indeed found that there’s no significant correlation between TC, LDLC, and TG and all-cause mortality in the 60–80 age group, but for the 80–100 age group, LDL-C and TG levels played a protective role, indicating the importance of maintaining proper LDL-C levels and higher TG levels to reach longevity. In this sense, our results imply that tea con-sumption may be unfavorable for lipid metabolism-related cardiovascular disease in older adults, but beneficial for longevity.

Another possible explanation could be the paradoxical effect of biological risk factors versus disease incidence. Just like the paradox effect between coffee drinking and coronary heart disease, proposed by van Dam[37], such an effect could also exist in the relationship tween tea consumption and CVD-related biomarkers. Firstly, the acute effects of tea drinking differs from the long-term effects of habitual tea consumption; Secondly, the physiological effects of tea drinking may depend on the type of tea consumed, not necessarily the same as for polyphenols in isolation; Thirdly, tea con-sumption may have beneficial effects on other biological pathways implicated in the development of CVD that could compensate for adverse effects. Moreover, a few RCT studies have already indicated that tea consumption does not significantly alter the concentrations of TC, LDL-C, HDL-C and TG concentrations[17–19], and high consumption of tea may contribute to several side effects due to its caffeine content[38], which may lead to increased serum lipid levels[39, 40]. The possible confounding effects should be also taken into account in the future studies by adding related variables such as the amount of tea consumptions

CRP is an important biomarker to predict the incident of CVD[41]. In our study, the longitudinal analyses found that occasional tea drinking was associated with lower hs-CRP changes in the 80–89 age group. Cross-sectional analyses by age and gender also found that black tea drinking at present was associated lower hs-CRP in females and in the 90–105 age group, and scented tea drinking at around age 60 was associated with lower hs-CRP in age 8089 group. These results partially converge with the pre-vious literature [42]. Both RCT and cohort studies showed that chronic tea drinking reduces C-reactive protein in healthy men[43, 44], and it is evidenced that black tea can significantly reduce CRP levels in both moderate and high risk groups compared to controls suggesting an anti-inflammatory action[45]. In this study, however, we didn’t find a significant correlation between green tea and white tea with CRP levels, which has been supported by some studies[46]. Whereas, our findings are indeed in line with recent systematic reviews and Meta-analyses. de Oliveira Assis et al. (2024), for exam-ple, proposed that green tea significantly decreased TNF-a levels but did not affect CRP levels[47]. Serban et al. (2015) found that green tea had no effect on plasma CRP con-centrations in the general population[48]. Haghighatdoost and Hariri (2019) summa-rized that green tea significantly affect other inflammatory markers, but could not significantly decrease serum CRP levels [49].

The effect variation of different teas on CRP may be related to their degree of fermentation. The main beneficial bioactive compounds in tea are polyphenols, bioa-vailability of which can be influenced by absorption, metabolism, distribution, and excretion in the body[50]. Previous study found that the absorption rate of black tea polyphenols is higher than that of green tea polyphenols[51], indicating that fermen-tation could significantly increase the bioavailability of fermented tea (black tea) as compared with unfermented or less fermented counterpart (green and white tea). As for scented tea, which is manufactured from semi-finished tea (such as black tea, white tea, green tea, etc.), its effect may inherit from the base tea[52, 53]. The different bioac-tive compounds in various types of tea could give us another clue. The main flavonoids are catechins in green tea. During the production of black tea, most catechins are oxidized to condensed flavonoids, such as theaflavins and thearubigins[8,50], recently shown to be a novel anti-inflammatory compound[54].

In the present study, the interaction analysis identified that tea consumption sig-nificantly modified the association of PRS with four CVD-related biomarkers: HDLC, LDLC, TG and hs-CRP. These results are not entirely consistent with previous study, which found that the associations between consumption of tea and blood lipids were not modified by genetic variants [6]. This study focused on single-nucleotide poly-morphisms (SNPs) affecting caffeine metabolism. Yet we constructed PRS for four CVD-related biomarkers, based on SNPs obtained from CLHLS genomewide associa-tion study (GWAS) and US. Health and Retirement Survey(HRS)GWAS datasets. SNPs per se typically have small effects and limited predictive power, whereas PRSs are more powerful[55]. Moreover our results are supported by a recent animal experi-mental study, which reported that green tea-derived Epigallocatechin-3-Galatte (EGCG) supplementation led to a decreased DNA methylation in mice at high risk for heart disease[56].

We also found that gender plays an important role in the relationship between tea consumption and CVD biomarkers in the older population. Gender-stratified analyses showed that tea consumption increase TG and LDLC levels, and decreased HDLC levels exclusively in older males, but has no significant effect in older females. Tea consumption occasionally only increased HDLC and Hemoglobin levels in older fe-males, but not older males. These results were consistent with previous findings. A case-control study, for instance, found that tea consumption is associated with a re-duced risk of CHD in female but not male populations in Guangzhou[57]. Another study reported that green tea drinking for more than 30 years was significantly related to reduced CHD risks in males; but in females, it was drinking green tea for more than 3 cups per day that could significantly reduce the CHD risk[58].

The current study demonstrated the complicated role of tea consumption in af-fecting CVD biomarkers and the interesting interactions between tea drinking and genetic variants. To the best of our knowledge, it is the first to investigate this topic in the Chinese population. Strengths of this study include the large sample size, the long follow-up period and the large number of follow-up assessments. Besides, we used PRSs based on GWAS to integrate the effects of multiple genetic loci, indicating the polygenetic effect rather than single SNP. Nevertheless, several limitations need to be considered. First, the study was based on self-reported data on tea consumption and thus likely introduced measurement errors. Second, given the observational nature of the study, it is possible that the associations as observed could be affected by hidden confounding factors. We think the mixed effect of tea consumption on CVD biomarkers and the interaction effect with genetic variants in this process deserve more future well-designed genetic-epidemiological studies, which are encouraged to incorporate more related covariates and to address the inconsistencies as discussed in this study.

## 5. Conclusions

The current study found that tea consumption had both beneficial and unfavora-ble effects on cardiovascular biomarkers in the older population. Specifically, it is protective against inflammatory related biomarkers and harmful for lipid metabolism related biomarkers. And these effects were all modified by genetic variants affecting lipid and inflammation metabolism. In addition, the association of tea consumption and genetic variants on CVD biomarkers varied by gender and age group. Our study suggested that the effects of drinking tea on the cardiovascular system are complicated, involving multiple physiological processes and metabolic pathways. When assessing the potential impacts of tea drinking for the heart health at older ages and developing related interventions in lifestyle, therefore, a more comprehensive scheme is needed to pay attention to the multifaceted nature of the tea drinking impact.

## Supplementary Material

Supplementary Files

This is a list of supplementary files associated with this preprint. Click to download.

• Supplementaryinformation.docx

## Figures and Tables

**Table 1:** Characteristics of the study samples, CLHLS 2008–2018

Characteristics	2008	2011	2014	2018	Min	Max
	N = 1,316	n = 2,303	n = 2,409	n = 2,475		
**Biomarker indicators**						
PG, mmol/L	5.34(1.35)	4.52(1.50)	5.25(1.39)	5.55(2.37)	0.02	42
TC, mmol/L	3.49(1.25)	4.25(0.94)	4.73(0.95)	5.12(4.70)	0.07	69.2
TG, mmol/L	1.46(1.04)	0.94(0.50)	1.20(0.59)	1.40(0.79)	0.01	6.13
Hs-CRP, mg/l	4.48(7.12)	2.95(5.77)	2.87(5.20)	3.02(6.14)	0	46.6
HDLC, mmol/L	1.17(0.31)	1.27(0.34)	1.39(0.35)	1.40(0.38)	0.01	2.49
Hemoglobin, g/L	126.6(23.5)	122.5(21.7)	126.1(18.4)	131.9(20.2)	1.03	188
LDLC, mmol/L	2.02(0.70)	2.52(0.77)	--	--	0.2	4.86
**Tea drinking behaviors**						
**Frequencies of tea drinking at present**						
Drink tea everyday, n(%)	253(20.5)	383(18.1)	362(15.5)	314(13.3)	0	1
Drink tea occasionally, n(%)	171(13.8)	404(19.1)	395(17.0)	160(6.8)	0	1
Drink tea rarely/never, n(%)	811(65.7)	1333(62.9)	1573(67.5)	1895(80.0)	0	1
**Frequencies of tea drinking at around age 60**						
Drink tea everyday, n(%)	243(19.8)	362(16.9)	312(13.7)	275(12.1)	0	1
Drink tea occasionally, n(%)	193(15.7)	436(20.4)	420(18.4)	274(12.0)	0	1
Drink tea rarely/never, n(%)	792(64.5)	1341(62.7)	1552(68.0)	1725(75.9)	0	1
**Type of tea drinking at present**						
Not tea drinker, n(%)	--	--	1573(79.2)	1895(81.4)	0	1
Green tea, n(%)	--	--	208(10.5)	217(9.3)	0	1
White tea, n(%)	--	--	75(3.8)	36(1.5)	0	1
Red tea, n(%)	--	--	42(2.1)	83(3.6)	0	1
Scented tea, n(%)	--	--	86(4.3)	97(4.2)	0	1
**Type of tea drinking at around age 60**						
Not tea drinker, n(%)	--	--	1552(81.7)	1725(85.2)	0	1
Green tea, n(%)	--	--	141(7.4)	154(7.6)	0	1
White tea, n(%)	--	--	88(4.6)	30(1.5)	0	1
Red tea, n(%)	--	--	35(1.8)	47(2.3)	0	1
Scented tea, n(%)	--	--	82(4.3)	69(3.4)	0	1
**Covariates**						
Age, mean(SD)	87.7(11.6)	85.9(12.0)	86.0(10.9)	84.1(11.1)	65	105
Female, n (%)	754(61.1)	1174(54.2)	1271(54.2)	1233(51.4)	0	1
East China, n(%)	420(34.0)	1139(52.6)	1264(53.9)	1187(49.4)	0	1
North China, n(%)	415(33.6)	1174(54.2)	1258(53.7)	1526(63.6)	0	1
Living in rural area, n (%)	923(74.7)	1788(82.5)	1843(78.7)	1814(75.6)	0	1
Education: Illiterates, n(%)	876(70.9)	1362(62.9)	1498(63.9)	1280(53.3)	0	1
Elementary school, n(%)	289(23.4)	605(27.9)	648(27.7)	833(34.7)	0	1
Middle school or higher, n(%)	72(5.8)	221(10.2)	260(11.1)	350(14.6)	0	1
Married, n (%)	348(28.2)	798(37.8)	852(37.0)	1079(45.4)	0	1
Co-resident with children, n(%)	739(59.8)	1100(50.8)	1121(47.8)	1235(51.4)	0	1
Log of income per capita, mean(SD)	7.98(1.04)	8.53(1.47)	8.75(1.46)	8.89(1.69)	0	11.51
Ever smoked, n (%)	252(20.4)	363(17.2)	336(14.4)	423(17.7)	0	1
Drink Alcohol, n (%)	224(18.1)	324(15.3)	323(14.0)	419(17.6)	0	1
Regular exercise, n (%)	244(19.8)	320(15.5)	341(14.9)	486(20.5)	0	1

**Table 2 – 1.** Cross-sectional associations between frequencies of tea consumption at around age 60 and cardiometabolic biomarkers

		All samples	Males	Females	Age65–79	Age80–89	Age90–105
	n(%)	β(95% CI)	β(95% CI)	β(95% CI)	β(95% CI)	β(95% CI)	β(95% CI)
**1.PG, mmol/L**							
n		8,137	4,444	3,693	2645	2158	3334
Rarely/Never	5410(68.3)	0.00	0.00	0.00	0.00	0.00	0.00
Occasionally	1323(16.7)	0.03(−0.10,0.17)	−0.05(−0.24,0.14)	0.14(−0.05,0.32)	0.12(−0.11,0.36)	−0.17(−0.36,0.02)*	0.08(−0.17,0.32)
Every day	1183(14.9)	−0.10(−0.22,0.02)	−0.15(−0.33,0.03)	−0.05(−0.21,0.11)	0.03(−0.20,0.27)	−0.17(−0.39,0.05)	−0.18(−0.35,0.00)
**2) TC, mmol/L**							
n		8,146	4,432	3,714	2661	2166	3319
Rarely/Never	5410(68.3)	0.00	0.00	0.00	0.00	0.00	0.00
Occasionally	1323(16.7)	0.02(−0.07,0.11)	0.05(−0.07,0.18)	0.01(−0.12,0.14)	−0.01(−0.18,0.16)	−0.07(−0.25,0.12)	0.13(0.01,0.24)
Every day	1192(15.0)	0.16(−0.06,0.39)	0.30(−0.06,0.65)	0.00(−0.14,0.14)	0.40(−0.08,0.88)	−0.07(−0.31,0.17)	0.01(−0.11,0.13)
**3. TG, mmol/L**							
n		8,146	4,439	3707	2645	2161	3340
Rarely/Never	5410(68.3)	0.00	0.00	0.00	0.00	0.00	0.00
Occasionally	1325(16.7)	0.03(−0.01,0.08)	0.03(−0.04,0.09)	0.05(−0.02,0.12)	0.08(−0.02,0.18)	0.04(−0.06,0.13)	−0.01(−0.06,0.05)
Every day	1188(15.0)	0.07(0.02,0.13)	0.10(0.02,0.18)***	0.03(−0.05,0.11)	0.12(0.02,0.22)	0.08(−0.03,0.19)	−0.01(−0.08,0.05)
**4.hs-CRP, mg/l**							
n		7,336	4,019	3,317	2346	1865	3125
Rarely/Never	4888(68.7)	0.00	0.00	0.00	0.00	0.00	0.00
Occasionally	1202(16.9)	−0.17(−0.56,0.22)	−0.38(−0.89,0.14)	0.03(−0.57,0.63)	0.12(−0.52,0.76)	0.15(−0.63,0.93)	−0.61(−1.24,0.02)*
Every day	1028(14.4)	−0.07(−0.50,0.36)	−0.32(−0.86,0.23)	0.27(−0.42,0.97)	−0.14(−0.59,0.30)	0.26(−0.81,1.34)	−0.04(−0.88,0.81)
**5.HDLC, mmol/L**							
n		8,150	4,446	3,704	2652	2168	3330
Rarely/Never	5416(68.3)	0.00	0.00	0.00	0.00	0.00	0.00
Occasionally	1326(16.7)	0.00(−0.02,0.02)	−0.01(−0.05,0.02)	0.01(−0.02,0.05)	−0.04(−0.08,0.00)*	0.01(−0.03,0.06)	0.02(−0.01,0.06)
Every day	1184(14.9)	0.02(−0.01,0.05)	0.02(−0.02,0.06)	0.01(−0.03,0.05)	0.00(−0.04,0.04)	0.03(−0.02,0.08)	0.03(−0.02,0.08)
**6) Hemoglobin, g/L**							
n		8,103	4,445	3,658	2625	2149	3329
Rarely/Never	5387(68.3)	0.00	0.00	0.00	0.00	0.00	0.00
Occasionally	1308(16.6)	1.34(0.12,2.55)**	1.08(−0.61,2.77)	1.49(−0.20,3.18)	−0.80(−2.72,1.11)	3.15(0.93,5.38)***	2.10(0.16,4.04)**
Every day	1187(15.1)	−1.58(−2.91,−0.26)**	−1.48(−3.34,0.39)	−2.47(−4.31,−0.64)***	−2.09(−3.98,−0.20)**	−0.76(−3.34,1.83)	−2.16(−4.56,0.23)*
**7.LDLC, mmol/L**							
n		3,403	1,930	1,473	1034	844	1525
Rarely/Never	2133(63.3)	0.00	0.00	0.00	0.00	0.00	0.00
Occasionally	631(18.7)	0.12(0.05,0.19)***	0.14(0.05,0.23)***	0.09(−0.01,0.20)	0.15(0.03,0.27)**	0.06(−0.07,0.18)	0.14(0.03,0.24)**
Every day	604(17.9)	0.08(0.00,0.15)	0.07(−0.03,0.18)	0.06(−0.05,0.17)	0.08(−0.04,0.21)	0.12(−0.03,0.27)	0.02(−0.10,0.15)

*** p < 0.01,

** p < 0.05. 95%, CI in parentheses.

Notes: All models are adjusted for age, sex, region, rural/urban residence, education, marital status, co-residence with children, family income, smoking, alcohol consumption, physical exercise, and dummies of waves.

**Table 2 – 2.** Cross-sectional associations between frequencies of tea consumption at present and cardiometabolic biomarkers

		All samples	Males	Females	Age65–79	Age80–89	Age90–105
	n(%)	β(95% CI)	β(95% CI)	β(95% CI)	β(95% CI)	β(95% CI)	β(95% CI)
**1.PG, mmol/L**							
n		8,137	4,444	3,693	2645	2158	3334
Rarely/Never	5612(69.7)	0.00	0.00	0.00	0.00	0.00	0.00
Occasionally	1128(14.0)	0.04(−0.06,0.14)	0.02(−0.13,0.16)	0.07(−0.08,0.22)	0.24(0.05,0.43)**	−0.22(−0.41,−0.03)**	0.02(−0.14,0.17)
Every day	1306(16.2)	0.10(−0.04,0.25)	0.13(−0.09,0.35)	0.07(−0.08,0.23)	0.30(0.05,0.56)	−0.07(−0.28,0.14)	0.04(−0.22,0.29)
**2.TC, mmol/L**							
n		8,146	4,432	3,714	2661	2166	3319
Rarely/Never	5612(69.7)	0.00	0.00	0.00	0.00	0.00	0.00
Occasionally	1130(14.0)	0.23(−0.01,0.47)*	0.16(−0.08,0.41)	0.32(−0.11,0.75)	0.48(−0.05,1.01)*	0.16(−0.38,0.69)	0.04(−0.06,0.14)
Every day	1312(16.3)	0.17(0.00,0.34)**	0.22(−0.04,0.49)*	0.11(−0.01,0.23)*	0.32(−0.07,0.71)	0.05(−0.10,0.21)	0.08(−0.02,0.19)
**3.TG, mmol/L**							
n		8,146	4,439	3,707	2645	2161	3340
Rarely/Never	5614(69.7)	0.00	0.00	0.00	0.00	0.00	0.00
Occasionally	1128(14.0)	0.04(−0.01,0.08)	0.04(−0.02,0.10)	0.04(−0.03,0.10)	0.07(−0.01,0.15)*	0.07(−0.03,0.17)	−0.01(−0.06,0.04)
Every day	1311(16.3)	0.07(0.02,0.11)***	0.10(0.03,0.16)***	0.02(−0.04,0.09)	0.09(0.00,0.17)**	0.11(0.01,0.20)**	0.02(−0.04,0.08)
**4.hs-CRP, mg/l**							
n		7,336	4,019	3,317	2346	1865	3125
Rarely/Never	5088(70.2)	0.00	0.00	0.00	0.00	0.00	0.00
Occasionally	1016(14.0)	−0.09(−0.48,0.31)	−0.10(−0.61,0.41)	−0.06(−0.67,0.54)	−0.06(−0.57,0.46)	−0.11(−0.91,0.69)	0.03(−0.70,0.77)
Every day	1142(15.8)	−0.13(−0.51,0.25)	−0.06(−0.53,0.41)	−0.19(−0.82,0.44)	0.29(−0.11,0.69)	−0.24(−1.06,0.57)	−0.44(−1.19,0.31)
**5. HDLC, mmol/L**							
n		8,150	4,446	3,704	2652	2168	3330
Rarely/Never	5615(69.7)	0.00	0.00	0.00	0.00	0.00	0.00
Occasionally	1131(14.0)	0.01(−0.01,0.03)	0.00(−0.02,0.03)	0.02(−0.01,0.05)	−0.01(−0.04,0.02)	0.02(−0.03,0.06)	0.02(−0.02,0.05)
Every day	1312(16.3)	0.02(0.00,0.04)**	0.01(−0.02,0.04)	0.03(0.00,0.07)**	0.00(−0.03,0.03)	0.05(0.00,0.09)	0.03(−0.01,0.07)
**6.Hemoglobin, g/L**							
n		8,103	4,445	3,658	2625	2149	3329
Rarely/Never	5593(69.8)	0.00	0.00	0.00	0.00	0.00	0.00
Occasionally	1110(13.9)	1.25(0.18,2.32)**	0.49(−1.03,2.02)	1.77(0.31,3.24)**	0.35(−1.25,1.94)	2.64(0.62,4.66)***	1.05(−0.88,2.98)
Every day	1310(16.3)	0.79(−0.28,1.85)	−0.20(−1.64,1.25)	1.38(−0.23,2.99)	0.31(−1.27,1.89)	1.55(−0.51,3.61)	0.48(−1.49,2.46)
**7.LDLC, mmol/L**							
n		3,403	1,930	1,473	1034	844	1525
Rarely/Never	2145(63.9)	0.00	0.00	0.00	0.00	0.00	0.00
Occasionally	576(17.2)	0.09(0.03,0.16)***	0.14(0.05,0.23)***	0.04(−0.05,0.14)	0.13(0.01,0.25)**	0.02(−0.10,0.15)	0.08(−0.02,0.18)
Every day	634(18.9)	0.12(0.05,0.18)***	0.18(0.08,0.27)	0.04(−0.06,0.14)	0.14(0.03,0.25)**	0.23(0.09,0.37)***	0.03(−0.08,0.14)

*** p < 0.01,

** p < 0.05. 95%, CI in parentheses.

Notes: All models are adjusted for age, sex, region, rural/urban residence, education, marital status, co-residence with children, family income, smoking, alcohol consumption, physical exercise, and dummies of waves.

**Table 3 – 1.** Cross-sectional associations between types of tea consumption at around age 60 and cardiometabolic biomarkers

		All samples	Males	Females	Age65–79	Age80–89	Age90–105
	n(%)	β(95% CI)	β(95% CI)	β(95% CI)	β(95% CI)	β(95% CI)	β(95% CI)
**1.PG, mmol/L**							
n		4,309	2,326	1,983	1426	1216	1667
Not tea drinker	3271(83.5)	0.00	0.00	0.00	0.00	0.00	0.00
Green tea	296(7.6)	0.21(−0.14,0.56)	0.17(−0.33,0.67)	0.23(−0.13,0.59)	0.05(−0.33,0.43)	0.18(−0.36,0.72)	0.49(−0.37,1.36)
White tea	119(3.0)	−0.26(−0.60,0.09)	−0.34(−0.80,0.12)	−0.08(−0.57,0.42)	−0.10(−0.78,0.58)	−0.41(−1.00,0.18)	−0.44(−0.80,−0.09)**
Red tea	82(2.1)	0.07(−0.36,0.50)	−0.26(−0.80,0.28)	0.60(−0.12,1.33)	0.08(−0.65,0.81)	−0.40(−1.12,0.32)	0.41(−0.25,1.07)
Scented tea	149(3.8)	0.19(−0.09,0.47)	0.01(−0.34,0.36)	0.43(−0.06,0.93)*	0.02(−0.55,0.58)	−0.16(−0.53,0.21)	0.59(0.11,1.07)**
**2.TC, mmol/L**							
n		4,313	2,324	1,989	1435	1217	1661
Not tea drinker	3277(83.5)	0.00	0.00	0.00	0.00	0.00	0.00
Green tea	295(7.5)	0.36(−0.28,1.00)	0.65(−0.33,1.62)	−0.06(−0.30,0.19)	0.73(−0.57,2.03)	−0.27(−0.60,0.05)	0.22(0.03,0.42)**
White tea	118(3.0)	0.42(−0.71,1.54)	0.82(−0.86,2.51)	−0.33(−0.83,0.17)	0.80(−1.63,3.23)	0.03(−0.46,0.51)	0.05(−0.26,0.36)
Red tea	82(2.1)	−0.09(−0.39,0.22)	0.12(−0.29,0.52)	−0.13(−0.52,0.25)	−0.14(−0.73,0.46)	−0.11(−0.78,0.55)	0.01(−0.42,0.45)
Scented tea	151(3.8)	0.01(−0.19,0.22)	0.09(−0.15,0.32)	0.00(−0.41,0.40)	0.18(−0.17,0.52)	−0.16(−0.71,0.40)	−0.06(−0.37,0.26)
**3.TG, mmol/L**							
n		4,315	2,329	1,986	1431	1215	1669
Not tea drinker	3270(83.4)	0.00	0.00	0.00	0.00	0.00	0.00
Green tea	297(7.6)	0.05(−0.04,0.14)	0.06(−0.06,0.17)	0.05(−0.09,0.19)	0.08(−0.07,0.23)	0.03(−0.12,0.18)	0.07(−0.07,0.20)
White tea	118(3.0)	0.05(−0.08,0.19)	0.07(−0.10,0.25)	−0.02(−0.22,0.18)	0.05(−0.19,0.29)	0.04(−0.28,0.36)	0.03(−0.11,0.18)
Red tea	83(2.1)	0.02(−0.11,0.14)	−0.10(−0.25,0.06)	0.17(−0.04,0.39)	0.09(−0.13,0.31)	−0.02(−0.20,0.15)	−0.01(−0.22,0.20)
Scented tea	153(3.9)	0.12(−0.01,0.24)*	0.11(−0.04,0.27)	0.12(−0.10,0.35)	0.14(−0.07,0.34)	0.03(−0.23,0.28)	0.19(−0.01,0.38)*
**4.hs-CRP, mg/l**							
n		4,304	2,320	1,984	1432	1216	1656
Not tea drinker	3271(83.4)	0.00	0.00	0.00	0.00	0.00	0.00
Green tea	296(7.5)	−0.22(−0.83,0.38)	−0.09(−0.90,0.73)	−0.35(−1.19,0.48)	0.38(−0.41,1.18)	−0.68(−2.07,0.72)	−0.88(−2.05,0.30)
White tea	119(3.0)	−0.28(−1.26,0.71)	−0.90(−1.81,0.01)*	0.88(−1.28,3.03)	−0.06(−1.26,1.15)	−0.89(−2.16,0.37)	−0.30(−2.56,1.96)
Red tea	82(2.1)	0.63(−0.81,2.07)	1.35(−0.90,3.60)	−0.73(−1.64,0.17)	0.87(−1.22,2.97)	1.11(−2.67,4.90)	−0.79(−2.38,0.79)
Scented tea	153(3.9)	0.37(−0.66,1.40)	−0.18(−1.12,0.76)	1.52(−0.79,3.84)	0.34(−0.47,1.16)	−0.99(−1.98,−0.01)**	1.69(−1.11,4.48)
**5.HDLC, mmol/L**							
n		4,317	2,327	1,990	1434	1221	1662
Not tea drinker	3269(83.3)	0.00	0.00	0.00	0.00	0.00	0.00
Green tea	298(7.6)	−0.01(−0.06,0.03)	−0.03(−0.09,0.03)	0.02(−0.05,0.09)	−0.02(−0.09,0.05)	−0.01(−0.10,0.08)	0.01(−0.08,0.09)
White tea	119(3.0)	0.05(−0.03,0.12)	0.07(−0.03,0.16)	0.01(−0.11,0.13)	−0.02(−0.14,0.10)	0.12(−0.04,0.29)	0.10(−0.02,0.22)*
Red tea	84(2.1)	−0.03(−0.11,0.05)	0.01(−0.10,0.11)	−0.07(−0.19,0.04)	−0.01(−0.11,0.09)	−0.13(−0.28,0.02)*	0.06(−0.10,0.22)
Scented tea	154(3.9)	−0.05(−0.11,0.00)*	−0.04(−0.11,0.04)	−0.08(−0.17,0.01)*	−0.01(−0.12,0.09)	0.06(−0.03,0.15)	−0.19(−0.29,−0.08)***
**6.Hemoglobin, g/L**							
n		4,314	2,330	1,984	1436	1211	1667
Not tea drinker	3257(83.1)	0.00	0.00	0.00	0.00	0.00	0.00
Green tea	302(7.7)	−2.57(−4.93,−0.21)**	−5.28(−8.61,−1.94)***	1.06(−1.75,3.87)	−2.72(−6.01,0.57)	−0.89(−5.59,3.82)	−3.78(−8.31,0.75)
White tea	119(3.0)	−2.96(−6.10,0.19)*	−2.96(−6.90,0.98)	−2.63(−7.55,2.29)	−4.68(−9.57,0.20)*	−3.39(−10.51,3.72)	0.04(−4.46,4.54)
Red tea	85(2.2)	2.40(−0.93,5.73)	2.97(−1.07,7.00)	1.37(−4.49,7.23)	1.54(−2.69,5.77)	3.91(−1.31,9.13)	0.96(−7.53,9.45)
Scented tea	154(3.9)	1.08(−1.53,3.68)	1.16(−2.00,4.32)	0.18(−4.41,4.77)	−2.72(−6.92,1.48)	4.60(0.63,8.58)**	2.10(−3.25,7.45)

Notes: All models are adjusted for age, sex, region, rural/urban residence, education, marital status, co-residence with children, family income, smoking, alcohol consumption, physical exercise, and dummies of waves.

*** p < 0.01,

** p < 0.05,

* p < 0.1, 95% CI in parentheses.

**Table 3 – 2.** Cross-sectional associations between types of tea consumption at present and cardiometabolic biomarkers

		All samples	Males	Females	Age65–79	Age80–89	Age90–105
	n(%)	β(95% CI)	β(95% CI)	β(95% CI)	β(95% CI)	β(95% CI)	β(95% CI)
**1.PG, mmol/L**							
n		4,309	2,326	1,983	1426	1216	1667
Not tea drinker	3467(80.5)	1.00	1.00	1.00	1.00	1.00	1.00
Green tea	422(9.8)	0.25(−0.02,0.52)*	0.24(−0.13,0.62)	0.25(−0.11,0.61)	0.40(0.05,0.76)**	−0.12(−0.52,0.28)	0.36(−0.28,0.99)
White tea	110(2.6)	−0.09(−0.43,0.25)	−0.22(−0.63,0.20)	0.24(−0.33,0.80)	0.14(−0.46,0.74)	−0.41(−0.88,0.06)*	−0.35(−0.77,0.08)
Red tea	128(3.0)	0.35(−0.36,1.06)	0.34(−0.69,1.36)	0.36(−0.23,0.96)	0.81(−0.70,2.32)	−0.28(−0.90,0.35)	0.24(−0.29,0.76)
Scented tea	181(4.2)	0.46(−0.04,0.97)*	0.69(−0.05,1.44)*	0.06(−0.37,0.49)	0.85(−0.29,1.98)	−0.13(−0.44,0.18)	0.55(−0.11,1.22)
**2.TC, mmol/L**							
n		4,313	2,324	1,989	1435	1217	1661
Not tea drinker	3468(80.4)	1.00	1.00	1.00	1.00	1.00	1.00
Green tea	425(9.9)	0.30(−0.15,0.76)	0.18(−0.30,0.65)	0.56(−0.38,1.50)	0.34(−0.40,1.08)	0.40(−0.83,1.63)	0.17(0.01,0.33)**
White tea	111(2.6)	0.65(−0.57,1.87)	0.97(−0.75,2.68)	0.01(−0.41,0.42)	0.95(−1.29,3.19)	0.10(−0.38,0.58)	0.19(−0.13,0.52)
Red tea	125(2.9)	−0.08(−0.36,0.19)	−0.12(−0.45,0.22)	0.15(−0.25,0.56)	−0.11(−0.64,0.42)	−0.14(−0.59,0.31)	0.01(−0.32,0.34)
Scented tea	183(4.2)	0.43(−0.39,1.26)	0.03(−0.21,0.26)	1.22(−0.99,3.43)	1.07(−0.98,3.12)	−0.04(−0.46,0.38)	0.09(−0.21,0.38)
**3.TG, mmol/L**							
n		4,315	2,329	1,986	1431	1215	1669
Not tea drinker	3465(80.3)	1.00	1.00	1.00	1.00	1.00	1.00
Green tea	427(9.9)	0.06(−0.01,0.14)*	0.08(−0.01,0.17)*	0.03(−0.09,0.16)	0.09(−0.03,0.21)	0.07(−0.10,0.24)	0.07(−0.04,0.17)
White tea	110(2.5)	0.04(−0.10,0.18)	0.03(−0.15,0.21)	0.01(−0.21,0.24)	0.05(−0.18,0.29)	0.02(−0.28,0.33)	0.06(−0.13,0.24)
Red tea	129(3.0)	0.06(−0.08,0.20)	0.05(−0.14,0.25)	0.08(−0.12,0.28)	0.24(−0.05,0.52)	−0.14(−0.28,0.00)*	−0.01(−0.15,0.14)
Scented tea	183(4.2)	0.15(0.03,0.27)**	0.10(−0.02,0.22)*	0.25(0.01,0.49)**	0.20(−0.02,0.42)*	0.05(−0.17,0.27)	0.19(0.03,0.35)**
**4.hs-CRP, mg/l**							
n		4,304	2,320	1,984	1432	1216	1656
Not tea drinker	3472(80.4)	1.00	1.00	1.00	1.00	1.00	1.00
Green tea	427(9.9)	0.02(−0.57,0.62)	0.08(−0.63,0.79)	0.08(−0.99,1.15)	0.29(−0.37,0.95)	−0.66(−1.76,0.43)	0.26(−1.15,1.67)
White tea	109(2.5)	0.26(−0.82,1.34)	−0.51(−1.43,0.41)	2.02(−0.68,4.73)	0.42(−0.72,1.56)	−1.21(−2.56,0.14)*	1.78(−1.52,5.07)
Black tea	127(2.9)	0.17(−0.99,1.34)	0.93(−0.83,2.69)	−1.27(−1.83,−0.71)***	0.56(−0.97,2.09)	0.59(−2.48,3.67)	−1.39(−2.44,−0.34)***
Scented tea	182(4.2)	0.45(−0.51,1.40)	0.91(−0.41,2.23)	−0.37(−1.60,0.85)	0.33(−0.34,1.01)	0.04(−1.66,1.74)	1.09(−1.26,3.44)
**5.HDLC, mmol/L**							
n		4,317	2,327	1,990	1434	1221	1662
Not tea drinker	3465(80.3)	1.00	1.00	1.00	1.00	1.00	1.00
Green tea	431(10.0)	0.01(−0.03,0.04)	−0.01(−0.05,0.04)	0.03(−0.03,0.10)	0.00(−0.05,0.06)	−0.01(−0.08,0.06)	0.02(−0.05,0.09)
White tea	108(2.5)	0.07(0.00,0.14)*	0.08(−0.01,0.16)*	0.04(−0.08,0.16)	0.01(−0.11,0.12)	0.14(−0.01,0.29)*	0.14(0.02,0.26)**
Red tea	129(3.0)	−0.02(−0.08,0.04)	−0.05(−0.12,0.02)	0.05(−0.06,0.15)	−0.01(−0.11,0.09)	−0.02(−0.13,0.09)	0.00(−0.11,0.12)
Scented tea	183(4.2)	−0.03(−0.09,0.03)	−0.03(−0.10,0.05)	−0.04(−0.13,0.05)	−0.02(−0.13,0.08)	−0.01(−0.10,0.09)	−0.08(−0.19,0.02)
**6.Hemoglobin, g/L**							
n		4,314	2,330	1,984	1436	1211	1667
Not tea drinker	3461(80.2)	1.00	1.00	1.00	1.00	1.00	1.00
Green tea	431(10.0)	−1.07(−2.85,0.71)	−3.29(−5.72,−0.86)***	2.08(−0.41,4.58)	−1.21(−4.00,1.57)	0.15(−3.01,3.30)	−1.96(−5.38,1.46)
White tea	110(2.5)	−0.54(−3.84,2.76)	−0.92(−4.76,2.92)	−0.45(−6.68,5.78)	−0.65(−5.83,4.54)	−3.31(−9.41,2.79)	1.73(−3.32,6.79)
Red tea	131(3.0)	4.15(1.64,6.66)***	3.23(0.09,6.37)**	5.15(0.96,9.33)**	3.16(−0.65,6.96)	4.98(0.76,9.19)**	4.91(−0.28,10.10)*
Scented tea	180(4.2)	1.73(−0.67,4.13)	1.02(−1.98,4.02)	2.52(−1.50,6.55)	0.18(−2.97,3.33)	3.43(0.03,6.83)**	2.36(−3.49,8.20)

All models are adjusted for age, sex, region, rural/urban residence, education, marital status, co-residence with children, family income, smoking, alcohol consumption, physical exercise, and dummies of waves.

*** p < 0.01,

** p < 0.05,

* p < 0.1, 95% CI in parentheses.

**Table 4 – 1.** Longitudinal associations of frequencies of tea consumption at around age 60 and changes of cardiometabolic biomarkers

		All samples	Males	Females	Age65–79	Age80–89	Age90–105
	n(%)	β(95% CI)	β(95% CI)	β(95% CI)	β(95% CI)	β(95% CI)	β(95% CI)
**1.PG, mmol/L**							
n		2,859	1,436	1,423	1237	863	759
Rarely/Never	1829(64.7)	1.00	1.00	1.00	1.00	1.00	1.00
Occasionally	526(18.6)	0.03(−0.12,0.19)	0.09(−0.11,0.29)	−0.01(−0.24,0.23)	−0.18(−0.53,0.17)	0.14(−0.17,0.45)	−0.06(−0.36,0.25
Every day	472(16.7)	0.04(−0.12,0.20)	0.10(−0.10,0.31)	−0.05(−0.28,0.17)	0.13(−0.15,0.40)	0.29(−0.03,0.61)	−0.28(−0.69,0.13
**2.TC, mmol/L**							
n		2,865	1,424	1,441	1245	866	754
Rarely/Never	1831(64.7)	1.00	1.00	1.00	1.00	1.00	1.00
Occasionally	526(18.6)	−0.06(−0.15,0.02)	−0.10(−0.22,0.02)	−0.01(−0.13,0.12)	−0.09(−0.23,0.05)	−0.04(−0.19,0.10)	−0.06(−0.22,0.09
Every day	474(16.7)	0.04(−0.26,0.35)	0.13(−0.32,0.59)	−0.12(−0.28,0.05)	0.20(−0.34,0.73)	−0.26(−0.47,−0.05)**	−0.09(−0.32,0.15
**3.TG, mmol/L**							
n		2,859	1,425	1,434	1235	864	760
Rarely/Never	1826(64.6)	1.00	1.00	1.00	1.00	1.00	1.00
Occasionally	523(18.5)	0.03(−0.04,0.09)	0.04(−0.05,0.13)	−0.01(−0.09,0.08)	0.07(−0.04,0.19)	0.02(−0.09,0.12)	0.00(−0.08,0.09
Every day	476(16.8)	0.01(−0.07,0.09)	0.00(−0.09,0.10)	0.06(−0.07,0.20)	0.00(−0.11,0.12)	−0.04(−0.18,0.11)	0.05(−0.07,0.16
**4.hs-CRP, mg/l**							
n		2,420	1,223	1,197	1023	703	694
Rarely/Never	1548(64.9)	1.00	1.00	1.00	1.00	1.00	1.00
Occasionally	454(19.0)	0.02(−0.68,0.71)	−0.01(−0.77,0.75)	0.02(−1.28,1.32)	1.08(0.14,2.02)**	−1.37(−2.69,−0.04)**	−0.10(−1.93,1.72
Every day	385(16.1)	0.27(−0.49,1.03)	0.23(−0.71,1.16)	0.17(−1.13,1.47)	1.03(−0.02,2.08)*	0.02(−0.74,0.78)	−1.11(−2.98,0.75
**5.HDLC, mmol/L**							
n		2,856	1,428	1,428	1234	866	756
Rarely/Never	1826(64.7)	1.00	1.00	1.00	1.00	1.00	1.00
Occasionally	524(18.6)	−0.02(−0.05,0.00)	−0.04(−0.08,0.00)**	0.01(−0.04,0.05)	−0.06(−0.10,−0.02)***	0.03(−0.02,0.07)	−0.02(−0.09,0.04
Every day	472(16.7)	−0.03(−0.06,0.00)**	−0.03(−0.07,0.01)	−0.02(−0.07,0.03)	−0.03(−0.07,0.00)*	−0.06(−0.12,0.00)**	0.00(−0.08,0.08
**6.Hemoglobin, g/L**							
n		2,767	1,392	1,375	1190	837	740
Rarely/Never	1769(64.6)	1.00	1.00	1.00	1.00	1.00	1.00
Occasionally	503(18.4)	0.29(−0.94,1.51)	−0.22(−1.87,1.44)	0.29(−1.52,2.09)	1.18(−0.60,2.97)	−1.29(−4.05,1.47)	1.24(−2.13,4.61
Every day	466(17.0)	0.29(−1.15,1.73)	0.82(−1.08,2.71)	0.18(−2.23,2.58)	0.29(−1.68,2.26)	0.08(−3.14,3.31)	−0.24(−4.04,3.55

*** p < 0.01,

** p < 0.05,

* p < 0.1, 95% CI in parentheses.

Notes: All models are adjusted for age, sex, region, rural/urban residence, education, marital status, co-residence with children, family income, smoking, alcohol consumption, physical exercise, and dummies of waves.

**Table 4 – 2.** Longitudinal associations of frequencies of tea consumption at present and changes of cardiometabolic biomarkers

		All samples	Males	Females	Age65–79	Age80–89	Age90–105
	n(%)	β(95% CI)	β(95% CI)	β(95% CI)	β(95% CI)	β(95% CI)	β(95% CI)
**1.PG, mmol/L**							
n		2,859	1,436	1,423	1237	863	759
Rarely/Never	1797(63.4)	1.00	1.00	1.00	1.00	1.00	1.00
Occasionally	515(18.2)	0.09(−0.09,0.26)	0.11(−0.14,0.35)	0.09(−0.17,0.36)	−0.11(−0.50,0.28)	0.23(−0.11,0.57)	−0.13(−0.51,0.2
Every day	524(18.5)	0.05(−0.12,0.22)	0.14(−0.08,0.36)	−0.07(−0.31,0.18)	0.22(−0.15,0.59)	0.26(−0.07,0.59)	−0.16(−0.56,0.2
**2.TC, mmol/L**							
n		2,865	1,424	1,441	1245	866	754
Rarely/Never	1800(63.4)	1.00	1.00	1.00	1.00	1.00	1.00
Occasionally	515(18.1)	−0.03(−0.16,0.10)	−0.10(−0.30,0.10)	0.06(−0.09,0.21)	−0.13(−0.38,0.12)	0.13(−0.04,0.30)	0.03(−0.15,0.2
Every day	526(18.5)	−0.07(−0.20,0.07)	−0.16(−0.40,0.08)	0.01(−0.15,0.17)	−0.08(−0.33,0.16)	−0.14(−0.32,0.04)	−0.05(−0.27,0.1
**3.TG, mmol/L**							
n		2,859	1,425	1,434	1235	864	760
Rarely/Never	1795(63.3)	1.00	1.00	1.00	1.00	1.00	1.00
Occasionally	511(18.0)	0.01(−0.06,0.07)	−0.01(−0.10,0.08)	0.03(−0.07,0.12)	0.02(−0.08,0.12)	−0.01(−0.14,0.12)	0.06(−0.03,0.1
Every day	529(18.7)	−0.01(−0.09,0.07)	−0.03(−0.13,0.08)	0.00(−0.10,0.10)	−0.03(−0.14,0.09)	−0.05(−0.19,0.10)	0.03(−0.08,0.1
**4.hs-CRP, mg/l**							
n		2,420	1,223	1,197	1023	703	694
Rarely/Never	1516(63.2)	1.00	1.00	1.00	1.00	1.00	1.00
Occasionally	451(18.8)	0.56(−0.22,1.34)	0.32(−0.59,1.23)	0.82(−0.56,2.19)	1.18(0.16,2.20)**	−0.31(−1.62,0.99)	0.31(−1.69,2.3
Every day	430(17.9)	0.07(−0.58,0.72)	−0.10(−0.86,0.66)	0.18(−1.03,1.38)	0.33(−0.44,1.10)	−0.55(−1.45,0.34)	0.08(−1.86,2.0
**5.HDLC, mmol/L**							
n		2,856	1,428	1,428	1234	866	756
Rarely/Never	1794(63.3)	1.00	1.00	1.00	1.00	1.00	1.00
Occasionally	515(18.2)	−0.03(−0.06,0.00)*	−0.03(−0.07,0.01)	−0.01(−0.06,0.04)	−0.02(−0.06,0.02)	−0.03(−0.09,0.03)	−0.02(−0.09,0.0
Every day	523(18.5)	−0.04(−0.07,−0.01)**	−0.04(−0.08,0.00)**	−0.02(−0.07,0.03)	−0.02(−0.06,0.02)	−0.09(−0.14,−0.04)***	−0.02(−0.09,0.0
**6.Hemoglobin, g/L**							
n		2,767	1,392	1,375	1190	837	740
Rarely/Never	1729(63.1)	1.00	1.00	1.00	1.00	1.00	1.00
Occasionally	493(18.0)	−0.52(−1.92,0.87)	−0.20(−2.07,1.66)	−0.61(−2.63,1.41)	0.21(−1.70,2.13)	−1.39(−4.51,1.74)	0.71(−2.86,4.2
Every day	520(19.0)	−0.81(−2.25,0.63)	0.51(−1.38,2.39)	−2.71(−4.83,−0.60)**	−0.39(−2.46,1.68)	−1.41(−4.60,1.78)	−1.40(−4.73,1.9

**Table 5 a.** Interaction between genetics and tea consumption on High-density lipoprotein cholesterol, mmol/L (HDLC)

	Main effect of PRS	Main effect of Tea drinking	Interaction	Pseudo R2
**1. Tea drinking around age 60**				
1) Whole samples	0.01(−0.01,0.04)	0.00(−0.04,0.04)	−0.05(−0.09,−0.01)***	0.044
2) Males only	0.02(−0.01,0.06)	−0.04(−0.09,0.01)	−0.05(−0.10,0.00)**	0.075
3) Females only	0.01(−0.02,0.04)	0.03(−0.03,0.08)	−0.05(−0.10,0.00)*	0.024
4) Samples aged 65–79 year	0.03(−0.02,0.08)	−0.09(−0.16,−0.02)**	−0.04(−0.11,0.03)	0.135
5) Samples aged 80–89 year	0.00(−0.05,0.05)	0.04(−0.04,0.12)	−0.08(−0.15,0.00)**	0.087
6) Samples aged 90–105 year	0.01(−0.02,0.04)	0.03(−0.02,0.09)	−0.04(−0.10,0.01)*	0.024
**2. Tea drinking at present**				
1) Whole samples	0.02(0.00,0.04)*	0.01(−0.02,0.05)	−0.06(−0.10,−0.03)***	0.047
2) Males only	0.03(−0.01,0.06)	−0.02(−0.07,0.04)	−0.05(−0.10,0.00)**	0.074
3) Females only	0.02(−0.01,0.05)	0.03(−0.02,0.08)	−0.07(−0.12,−0.02)***	0.026
4) Samples aged 65–79 year	0.05(0.00,0.09)*	−0.07(−0.13,0.00)*	−0.06(−0.13,0.00)*	0.138
5) Samples aged 80–89 year	0.02(−0.03,0.07)	0.05(−0.03,0.12)	−0.11(−0.18,−0.03)***	0.096
6) Samples aged 90–105 year	0.01(−0.01,0.04)	0.04(−0.02,0.09)	−0.05(−0.10,0.00)*	0.024

Notes: Covariates include: age, east/middle/west regions, rural/urban residence, education, marital status, co-residence with children, smoking, drinking alcohol, and exercise regularly.

*** p < 0.01,

** p < 0.05,

* p < 0.1, 95% CI in parentheses.

Notes: All models are adjusted for age, sex, region, rural/urban residence, education, marital status, co-residence with children, family income, smoking, alcohol consumption, physical exercise, and dummies of waves.

## Data Availability

All data used in this study were stored at http://opendata.pku.edu.cn and available upon request.
